# Effectiveness of a 3-year community-based intervention for blood pressure reduction among adults: a repeated cross-sectional study with a comparison area

**DOI:** 10.1038/s41371-022-00672-2

**Published:** 2022-04-08

**Authors:** Rim Ghammam, Jihene Maatoug, Imed Harrabi, Sihem Ben Fredj, Nawel Zammit, Tiina Laatikainen, Erkki Vartiainen, Dinesh Neupane, Hassen Ghannem

**Affiliations:** 1 Université de Sousse, Faculté de Médecine de Sousse, 4000, Sousse, Tunisie; 2 Hôpital Farhat Hached, Service d’Epidémiologie, «LR19SP03», 4000, Sousse, Tunisie; 3 National Institute for Health and Welfare (THL), Helsinki, Finland; 4 Department of Epidemiology, Johns Hopkins Bloomberg School of Public Health, Johns Hopkins University, Baltimore, MD USA

**Keywords:** Hypertension, Disease prevention, Lifestyle modification, Preventive medicine

## Abstract

Raised blood pressure is the leading risk factor for cardiovascular diseases. We aimed to demonstrate the effectiveness of a 3-year community-based healthy lifestyle promotion intervention at a neighbourhood level for blood pressure reduction among adults in the context of a political transition. We conducted repeated cross-sectional surveys including 1880 (940 from each area) participants at baseline and 1977 (1001 in the intervention area; 976 comparison area) participants at follow-up. Data collection was conducted through home visits. Multiple linear regression models were used to identify the intervention effect and factors associated with blood pressure change in each area. The prevalence of hypertension was 4.4% lower (35.8% vs. 31.4%) in the intervention area after 3 years (*p* = 0.044). The mean systolic and diastolic blood pressures changed significantly in the intervention area, from 132.4 ± 19.2 mmHg at baseline to 130.6 ± 17.7 mmHg at follow-up (*p* = 0.035) and from 78.7 ± 11.8 mmHg to 76.9 ± 11.1 mmHg (*p* < 10^−3^), respectively. In the control group, the mean systolic blood pressure increased from 129.4 ± 17.8 mmHg to 130.4 ± 17.9 mmHg (*p* = 0.38). A significant protective effect of the intervention on systolic (β = −0.4; 95% CI: −0.76; −0.06) and diastolic blood pressures (β = −0.22; 95% CI: −0.38; −0.07) was found in the intervention area. In the control area, the effect was not significant for systolic blood pressure. Lifestyle intervention at the neighbourhood level, in the context of a sociopolitical transition, was found to be effective for reducing blood pressure in Sousse, Tunisia. This approach could be scaled up and applied in other similar settings. Future research also needs to focus on designing, implementing, and evaluating multisectoral action plans and legislative measures.

## Introduction

Cardiovascular diseases (CVDs) are responsible for nearly 30% of deaths worldwide [1]. Globally, 51% of stroke and 45% of ischaemic heart disease deaths are attributable to high systolic blood pressure (SBP) [1]. In 2019, the number of people aged 30–79 years with hypertension was estimated to be almost doubled compared to that in 1990 [2].

At least three-quarters of deaths from CVDs worldwide occur in low- and middle-income countries (LMICs) [3]. Many lifestyle factors contribute to the high prevalence of hypertension, including high-salt and high-fat diets, inadequate intake of fruits and vegetables, overweight and obesity, harmful alcohol use, physical inactivity, psychological stress, socioeconomic determinants, and inadequate access to health care [2]. People in LMICs often have limited access to effective and equitable health care services [3].

The concept of community control was introduced in the World Health Organization’s (WHO) cardiovascular program in the late 1960s with a special focus on hypertension [4]. Thirty years after its launch, the North Karelia project from which our study was inspired succeeded in decreasing mean systolic blood pressure by 15 mmHg among men and by 24 mmHg among women. Mean diastolic blood pressure decreased by 8 and 14 mmHg among men and women, respectively [5]. There is strong evidence of the health benefits of lowering blood pressure through population-wide and individual interventions [2]. Cessation of tobacco use, reduction of salt in the diet, consumption of fruits and vegetables, regular physical activity, and avoidance of harmful alcohol use have been shown to reduce the risk of hypertension [3].

Health promotion and intervention programmes targeting different hypertension risk factorsresulted invarying levels of decrease in systolic blood pressure, ranging from 0.32 to 5.6 mmHg [6]. Many studies have confirmed that a modest population‐wide decrease in systolic blood pressure (1–2 mmHg) could have a significant impact on preventing CVD incidence [7].

Tunisia, a North African country, is now experiencing demographic and epidemiological transitions with a change from a burden of infectious diseases to a burden of chronic diseases [8]. In addition to the country’s sociocultural transition, there has beena shift from the consumption of a Mediterranean diet rich in fibre, fruits and vegetables to the consumption of a Western diet with increased consumption of fast food [8]. Epidemiological studies found that 30.6% of adults in Tunisia are hypertensive [9], and the proportion of people with modifiable risk factors is increasing [10]. The health system is struggling to manage chronic diseases with a shortage of funds in the National Health Insurance Funds at the primary care level [11]. The current national strategies and the recently published Tunisian guidelines mainly focus on treatments and not on population-based prevention and health promotion [12, 13].

To our knowledge, no other intervention studies aiming to promote a healthy lifestyle at the population level in Tunisia have been conducted. The most successful interventions have been implemented in developed countries [14], and there is no published evidence of the effectiveness of such programmes in the particular context of LMICs. In this study, we aimed to assess the effectiveness of a community-based healthy lifestyle promotion intervention for systolic and diastolic blood pressure reduction among adults in Tunisia in the context of a sociopolitical transition.

## Methods

### Study design

**Fig. 1:**
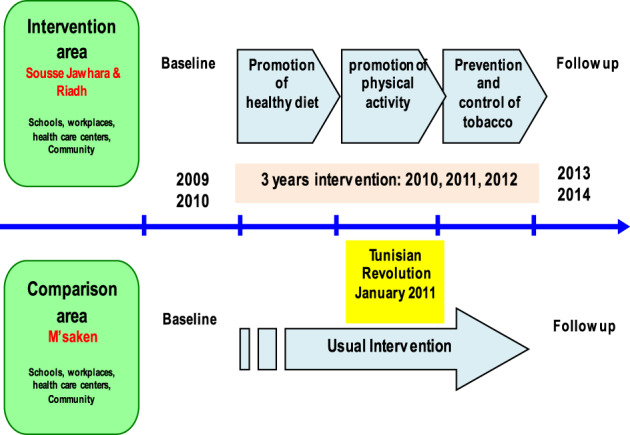
The design of the pre-post intervention study (repeated cross-sectional surveys): baseline and follow-up with a comparison area in the region of Sousse Tunisia, 2009–2014.

We conducted a repeat cross-sectional study in intervention and comparison areas. The intervention areas were located in Sousse Jawhra and Sousse Erriadh, whereas the comparison area was located in M’saken. We carried out a baseline survey in a group of adults aged 18–65 years in the region of Sousse in 2009–2010 before the intervention and a follow-up survey assessing the same parameters in 2013–2014 after the intervention using independent cross-sectional surveys in both areas (Fig. 1). The intervention lasted for 3 years. In the control area, there were no specific intervention programmes beyond usual governmental programmes. The intervention and comparison areas were 20 km apart but were similar in regard to demographics, environment, culture, and socioeconomic status.

### Sample size and study population

The sample size calculation was based on a type 1 error (α) of 5%, a type 2 error (β) of 20%, a change of 3 mmHg in systolic blood pressure between baseline and follow-up, and an intracluster correlation coefficient of 0.01 [6, 7, 15]. According to these values, the required sample size would be 292 in each group. Considering the clustering effect and 15% loss to follow-up, the sample size was readjusted to 920 individuals in each group.

**Fig. 2:**
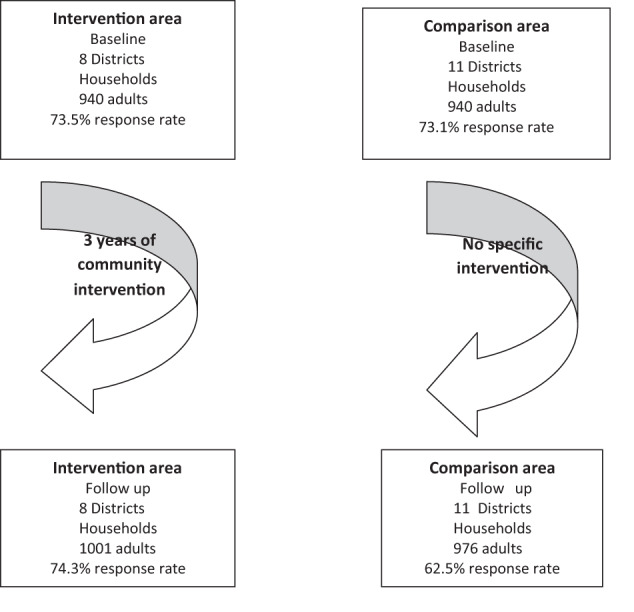
Consort diagram.

The region of Sousse in the centre of East Tunisia is divided into 15 delegations with 544,413 inhabitants and 124,519 households. The City of Sousse is the third largest city in Tunisia and is the capital of central Tunisia. The minimum sample size was selected based on the estimated number of districts and households from the last national census using data from the National Statistical Institute. We randomly selected 8 and 11 districts from the intervention and comparison areas, respectively. We selected 500 households in each area; all adults, aged 18–65 years old, present in the households on the day of data collection were included. We surveyed 940 participants for the baseline surveys in the intervention (73.5% response rate) and comparison areas (73.1% response rate) and 1001 and 976 participants for the follow-up surveys in the intervention (74.3% response rate) and comparison areas (62.5% response rate), respectively (Fig. 2). People were considered missing after at least two home visits, one of which was on a weekend.

### Data collection

We used a pretested Arabic questionnaire to collect data before and after the intervention. The questionnaire was translated by experts from the CIH Pilot study version 5 [16]. Through personal interviews, trained investigators collected information on sociodemographic characteristics, medical history, and attitudes and beliefs towards chronic disease risk factors.

Weight was measured to the nearest 0.1 kg using a calibrated portable electronic scale (BEUREUR). Height, with the participants in a standing position with bare feet, was measured to the nearest 0.5 cm. Blood pressure was measured by a health professional twice at rest using an adjustable cuff electronic sphygmomanometer (OMRON HEALTHCARE EOROPE B.V.Kruisweg 577-2132 NA Hoofddorp, The Netherlands, M3 Intellisense, (HEM-7051-E)). The first measurement was taken after 20 min of rest and the second after completing the questionnaire (15 min).

### Variable definitions and classification

The mean value of two measurements was used for systolic and diastolic blood pressures to define high blood pressure on the day of the measurement. Adults were considered hypertensive if they had systolic blood pressure ≥140 mmHg and/or diastolic blood pressure ≥90 mmHg [17]. We used the WHO classification of body mass index (BMI) to define overweight and obesity [18]; overweight was defined as a BMI ≥ 25, and obesity was defined as a BMI ≥ 30.

Physical activity was investigated using the International Physical Activity Questionnaire (IPAQ) [14]. The recommended level of physical activity (RPA) was established according to the WHO definition for adults (30 min of intense-to-moderate physical activity at least 5 days a week) [19]. Sedentary behaviour was defined as engaging in any seated activity for more than 2 h per day.

Smokers were participants who answered yes to the question“ Do you currently smoke tobacco products such as cigarettes, cigars, or water pipes?” We defined the recommended amount of fruits and vegetables (5 FV) as five or more servings per day [20].

Socioeconomic status (SES) was based on the asset index developed by the World Bank [21]. Factor analysis was used to give different weights to different household items and to develop a comprehensive asset index (first extracted component in the analysis), which was used as a proxy of SES. Then, the Ward method was used to obtain four hierarchical SES levels: low, low-middle, high-middle, and high.

### Intervention programme

This study is part of a comprehensive integrated community-based intervention programme for chronic disease risk factor prevention. According to the social-ecological model of health promotion and disease prevention, individuals’ behaviours and attitudes are influenced by the environments in which they live (family environment, community, society, micro- and macro-level factors) [17]. Thus, health promotion activities targeted at the neighbourhood level could have potential benefits. This paper reports the results from the neighbourhood intervention comparing population-level changes in blood pressure.

### Intervention delivery

The neighbourhood intervention programme consisted mainly of education sessions and open sensitisation days promoting physical activity, healthy diet, and tobacco control. There are no particular ethnic groups in Tunisia, and the eating behaviours of people in Tunisia are similar. Messages were delivered using flyers, radio jingles, radio sessions, and open sensitisation days. Specific messages during the education session targeted different risk factors:

- Physical activity: Written and oral messages were provided by educators to help participants integrate physical activity into their everyday life.
- Healthy eating: The main written and oral messages to promote healthy eating among participants were “Eat more fruits and vegetables and less fat, sugar, and salt,” “drink more water,” and “do not snack between meals.”
- Tobacco cessation: In addition to oral and written warning messages, smokers benefited from measuring the carbon monoxide in their exhaled air. The Fagerstrôm score was used to evaluate the degree of addiction. People willing to quit were referred to antismoking counselling.

The intervention programme consisted of open sensitisation days every last Sunday of the month in the neighbourhoods in front of the supermarkets. During these days, the programme team provided risk factor screening and education for persons at risk. People with health problems or those desiring to stop smoking were referred to appropriate counselling services. During the first and third years of the intervention, flyers containing various chronic disease preventive messages were distributed to all households belonging to the intervention districts as well as to participants on open sensitisation days. Various radio spots to promote a healthy diet, physical activity, and control of tobacco use were broadcasted three times daily for 6 months by local radio stations. Mass media intervention included more than ten radio sessions narrated by the project team to introduce the programme and a variety of specialists (e.g., paediatricians, endocrinologists, dietitians, physical activity teachers, family doctors, psychologists) to disseminate awareness messages about healthy eating habits, physical activity, and tobacco cessation.

A team of professionals in communication and health care designed educational materials specifically for the purposes of this study to be used in different settings. The materials included flyers, posters, videos, and radio spots containing various chronic disease preventive messages. Ten two-day training sessions to manage hypertension, diabetes, obesity, and tobacco cessation and to evaluate the cardiovascular risk for all doctors and paramedics in primary care centres of the intervention area were organised in collaboration with the Regional Health Directorate in Sousse (Ministry of Health) and the Faculty of Medicine of Sousse. Training sessions were facilitated by diverse project teams, including a cardiologist, dietician, doctors from the Regional Health Directorate in Sousse (responsible for the national programme of hypertension and diabetes), psychologist, and paediatrician. The team was trained to ensure standardisation of the educational messages.

### Data analysis

Statistical analysis was performed using SPSS 25.0 software. Although the majority of participants exhibited different results between the baseline and follow-up surveys, we assumed that participants were similar on various factors in both surveys except for blood pressure, as we applied the same sampling technique from the same geographical area for both surveys (Table 1).

**Table 1:** Sociodemographic characteristics and evolution of screened prevalence of adults participating in the study before and after the intervention in the intervention and comparison areas.

	Intervention area			Comparison area		
	Baseline	Follow-up	*p* value	Baseline	Follow-up	*p* value
Age M (SD) years	37.20 (13.22)	39.25 (13.61)	**0.001**	38.61 (13.73)	40.43 (13.96)	**0.004**
Age group *n* (%)			**0.007**			**0.036**
[18–29]	346 (37.0)	306 (30.6)		295 (31.5)	260 (26.7)	
[30–39]	173 (18.5)	206 (20.6)		214 (22.9)	206 (21.2)	
[40–49]	196 (21.0)	202 (20.2)		187 (22.6)	226 (23.2)	
[50–65]	219 (23.4)	287 (28.7)		240 (25.6)	281 (28.9)	
Sex *n* (%)			0.669			**0.010**
Male	406 (43.2)	442 (44.2)		271 (28.8)	335 (34.3)	
Female	534 (56.8)	559 (55.8)		669 (71.2)	641 (65.7)	
Educational level *n* (%)		**<0.001**			**0.001**	
Illiterate or primary	238 (25.3)	330 (38.8)		400 (42.6)	497 (51.0)	
College or secondary	430 (45.8)	501 (50.2)		368 (39.2)	341 (35.0)	
University	271 (28.9)	168 (16.8)		171 (18.2)	137 (14.1)	
Marital status *n* (%)			0.065			**0.018**
Not married	355 (37.9)	339 (33.9)		317 (33.8)	278 (22.7)	
Married	582 (62.1)	662 (66.1)		622 (66.2)	689 (71.3)	
Employment status *n* (%)		**<0.001**			**<0.001**	
Not working	82 (8.8)	156 (15.7)		92 (9.8)	143 (14.7)	
Worker	416 (44.4)	467 (46.9)		264 (28.1)	357 (36.7)	
Student	157 (16.8)	96 (9.6)		127 (13.5)	87 (8.9)	
Housewife	241 (25.7)	224 (22.5)		417 (44.5)	342 (35.1)	
Retired	41 (4.4)	53 (5.3)		38 (4.1)	45 (4.6)	
Socioeconomic level *n* (%)		**<0.001**			**<0.001**	
Low	46 (4.9)	18 (1.8)		19 (2.0)	35 (3.6)	
Low-middle	466 (49.6)	456 (45.6)		440 (46.8)	427 (43.8)	
High-middle	372 (39.6)	425 (42.5)		446 (47.4)	418 (42.8)	
High	56 (6.0)	102 (10.2)		35 (3.7)	96 (9.8)	
Screened hypertension *n* (%)	325 (35.8)	311 (31.4)	**0.044**	274 (29.3)	296 (30.3)	0.625

Statistically significant *p*-values are in bold.

Continuous variables are presented as the mean (standard deviation), and categorical variables are presented as percentages (%). The level of statistical significance was set at *p* < 0.05. Multiple linear regression models adjusting for cluster effects were used separately for intervention and comparison areas. The effect of the intervention (which was coded as pre-intervention or post-intervention), BMI, tobacco use, consumption of a recommended amount of fruits and vegetables daily, salt consumption, sedentary lifestyle, engagement in 30 min of physical activity daily at least 5 days a week (RPA), and use of hypertension medication were the variables included in the model. We adjusted for sociodemographic characteristics (sex, age, SES, and education level).

### Ethical considerations

This study was conducted with the consideration of the rights and integrity of the participants. Ethical clearance was obtained from the Ethical Committee of Farhat Hached University Hospital in Sousse, Tunisia. All participants gave informed consent. At the end of the study period, a delayed intervention was offered to the comparison area.

## Results

Figure 2 and Table 1 illustrate the response rates and sociodemographic characteristics of the independent cross-sectional samples in both the intervention and comparison areas.

The prevalence of hypertension decreased significantly in the intervention area, from 35.8% at baseline to 31.4% at follow-up (*p* = 0.04). Although the prevalence was higher at follow-up than at baseline (29.3% vs. 30.3%) in the comparison area, the difference was not statistically significant (*p* = 0.62).

**Fig. 3:**
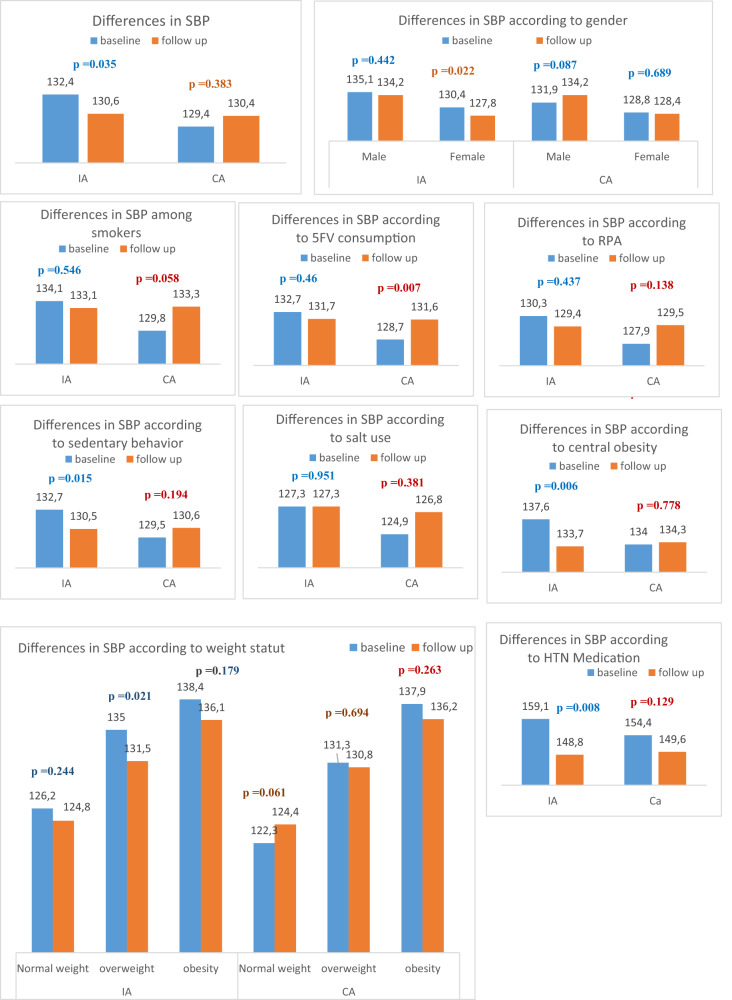
Systolic blood pressure: differences in systolic blood pressure (SBP) (mmHg) among adults before and after the intervention in the intervention area (IA) and comparison area (CA).

**Fig. 4:**
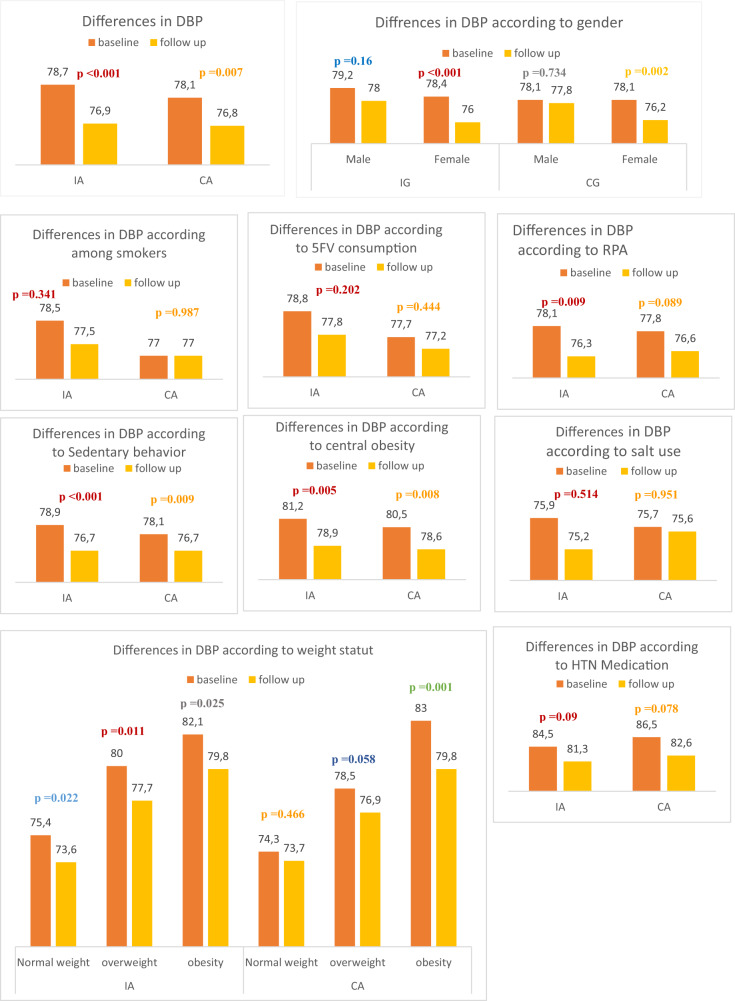
Diastolic blood pressure: differences in diastolic blood pressure (DBP) (mmHg) among adults before and after the intervention in the intervention area (IA) and comparison area (CA).

The mean systolic and diastolic blood pressures in the intervention area were significantly lower at follow-up than at baseline. After stratification according to sex, the association remained statistically significant only for women (SBP = 130.4 ± 19.9 mmHg to 120.6 ± 17.8 mmHg, *p* = 0.022; DBP = 78.7 ± 11.8 mmHg to 76.9 ± 11.1 mmHg, *p* < 0.001) (Figs. 3 and 4). A statistically significant association of diastolic blood pressure was also found for women in the comparison area. According to age group, the mean systolic and diastolic blood pressures differed among all age categories in the intervention area (Figs. 3 and 4, Supplementary Tables 1 and 3).

A significant difference in mean systolic and diastolic blood pressures was found only among nonsmoking adults in the intervention area (SBP: 131.9 ± 19.4 mmHg to 129.9 ± 17.4 mmHg, *p* = 0.04; DBP: 78.9 ± 11.5 mmHg to 76.7 ± 11, *p* < 0.001). In the comparison area, a significant difference was only found for diastolic blood pressure among nonsmokers (Figs. 3 and 4, Supplementary Table 2 and 4).

Regarding physical activity, mean systolic and diastolic blood pressures differed among adults who reported practising regular physical activity in the intervention area. This finding was significant only for diastolic blood pressure. In the comparison area, we observed a nonsignificant change in mean systolic and diastolic blood pressures in the same category (Figs. 3 and 4, Supplementary Tables 2 and 4).

Figures 3 and 4 show the evolution of systolic and diastolic blood pressures before and after the intervention in both groups, according to different risk factors. According to multiple linear regression models, factors associated with changes in systolic blood pressure in the intervention area were intervention effect (β = −0.405, *p* = 0.029), female sex (β = −0.801, *p* < 10^−3^), age (β = 0.045, *p* < 10^−3^), high SES (β = 0.488, *p* = 0.012), low or middle SES (β = 0.508, *p* = 0.022), BMI (β = 0.051, *p* < 10^−3^) and use of HTN medication (β = 1.482, *p* < 10^−3^). The intervention effect was associated with significantly lower systolic blood pressure in the intervention area. This effect was not significant in the comparison area (β = −0.1, *p* = 0.154) (Table 2).

**Table 2:** Factors associated with systolic and diastolic blood pressure changes among adults in the intervention and comparison areas after multiple linear regressions (results of 4 Models).

	Intervention area				Comparison area			
	Estimate	CI_95%_		*p* value	Estimate		CI_95%_	*p* value
Systolic blood pressure								
Time effect	−0.405	−0.756	−0.055	**0.029**	−0.100	−0.244	0.044	0.154
Women	−0.801	−0.980	−0.623	**<0.001**	−0.537	−0.659	−0.415	**<0.001**
Age (years)	0.045	0.041	0.048	**<0.001**	0.039	0.027	0.050	**<0.001**
Socioeconomic level								
High	0.488	0.147	0.829	**0.012**	−0.356	−1.109	0.396	0.316
High-middle	0.369	−0.097	0.836	0.103	−0.145	−0.788	0.498	0.627
Low-middle	0.508	0.097	0.919	**0.022**	−0.336	−0.897	0.224	0.211
Low	–	–	–	–	–	–	–	–
Education level								
University	−0.226	−0.711	0.258	0.306	−0.239	−0.523	0.045	0.090
College or secondary	−0.079	−0.442	0.284	0.622	−0.186	−0.322	−0.050	**0.012**
Illiterate or primary	–	–	–	–	–	–	–	–
BMI (kg/m^2^)	0.051	0.040	0.063	**<0.001**	0.046	0.029	0.062	**<0.001**
HTN medication	1.482	1.304	1.660	**<0.001**	1.492	1.094	1.889	**<0.001**
Intercept	10.283	9.558	11.009	**<0.001**	10.799	9.887	11.712	**<0.001**
Diastolic blood pressure								
Time effect	−**0.225**	−0.384	−0.066	**0.012**	−0.212	−0.321	−0.103	**0.001**
Women	−0.261	−0.335	−0.187	**<0.001**	−0.149	−0.247	−0.050	**0.007**
Age (years)	0.016	0.011	0.021	**<0.001**	0.012	0.005	0.020	**0.005**
Socioeconomic level								
High	−0.221	−0.821	0.379	0.413	0.133	−0.304	0.571	0.512
High middle	0.354	−10.023	0.315	0.251	0.189	−0.038	0.416	0.093
Low middle	−0.237	−0.838	0.363	0.381	0.152	0.032	0.273	**0.018**
Low	–	–	–	–	–	–	–	–
Education level								
University	−0.002	−0.183	0.180	0.982	−0.144	−0.331	0.043	0.118
College or secondary	0.018	−0.081	0.117	0.682	−0.127	−0.290	0.036	0.112
Illiterate or primary	–	−0.183	0.180	–	–	–	–	–
BMI (kg/m^2^)	0.033	0.025	0.041	**<0.001**	0.033	0.029	0.037	**<0.001**
HTN medication	0.264	0.115	0.413	**0.004**	0.147	−0.093	0.387	0.202
Intercept	6.772	5.892	7.653	**<0.001**	6.420	6.000	6.840	**<0.001**

Adjusted for cluster effect.

Statistically significant *p*-values are in bold

Independent factors associated with changes in diastolic blood pressure in the intervention area were intervention effect (β = −0.225, *p* = 0.012), female sex (β = −0.261, *p* < 10^−3^), age (β = 0.016, *p* < 10^−3^), BMI (β = 0.033, *p* ≤ 10^−3^) and use of HTN medication (β = 0.264, *p* = 0.004). The intervention effect was associated with significantly lower diastolic blood pressure in both the intervention and comparison areas(Table 2).

## Discussion

This was the first neighbourhood intervention implemented at the community level in Tunisia to reduce blood pressure at the population level. This study showed that a 3-year community-based intervention promoting a healthy lifestyle effectively resulted in a small but clinically significant blood pressure decrease.

The reduction in blood pressure was greater among women than among men. Many factors, such as physiology, immune response, and better hypertension awareness among women, may explain the sex difference in blood pressure [22]. However, some health intervention programmes also showed better results in men than in women [23]. Thus, future studies are needed to explore the motivating factors for adopting a healthy lifestyle in both sexes.

Studies including ours provide unequivocal evidence that lifestyle modifications to reduce body weight should be a major component in the prevention and control of hypertension [24, 25].Although documented by some research, we did not find a significant effect of SES on blood pressures [26]. Careful monitoring of hypertension and risk factors by socioeconomic group in LMICs, with a focus on the most vulnerable groups,is needed, as there is a paucity of evidence in this area [26].

A large sample size, follow-up data, and real-world implementation were strengths of this study. The limitations of the study were non randomisation, possible recall bias in self-reported data, and different participants for baseline and follow-up surveys. A longer follow-up duration would have been useful to explore the sustainability of the programme in the long run.

Although this project was conducted with a different target group, we cannot rule out the indirect effect of other interventions at workplaces, schools, and primary health care centres. Some of the results might be explained by regression to the mean. However, given the large sample size, this phenomenon likely only explains a small component of the observed changes. In addition, the comparison area did not serve as a true placebo group because they continued receiving existing government programmes. They had also received some on and off awareness interventions and might also have had access to certain interventions and broadcasting programmes disseminated by the media. In addition, we had good response rates in the intervention and control areas. All participants present in the randomly selected households at the time of the visit, whatever their level of compliance, were included. Therefore, selection bias due to subjects who had not complied with the intervention was minimised.

Despite these limitations, due to the large sample size and the use of regression analysis with adjustment, we do not expect too much deviation from the current results. Of course, prospective studies and cluster randomised trials are needed to establish cause and effect relationships in the future.

A stand-alone health education intervention at the community level was found to be effective in reducing blood pressure. However, it is also equally important to strengthen the capacity of individuals and populations to make healthier choices and follow lifestyle patterns that foster good health [27].

To our knowledge, our intervention implemented a unique strategy that targeted the whole population at the community level regardless of the risk level in the region. The health care strategy in Tunisia is essentially based on the high-risk approach. Combining the high-risk approach with a population-based approach for the management of hypertension and its risk factors will lead to greater effectiveness, hence the importance to scale up this programme. Health promotion interventions should engage state and non state actors within and outside the health sectors [27]. Future research is also needed to focus on designing, implementing, and evaluating multisectoral action plans and legislative measures.

### Summary table

#### What is known about topic

- Health promotion and intervention programmes targeting different hypertension risk factors are effective to decrease blood pressure

- Most successful experiences in the fight against CVD risk factors are coming from developed countries, and no evidence on the effectiveness of such programmes in a particular context of LMIC

#### What this study adds

- Health education intervention at the community level was found to be effective in reducing blood pressure in a LMIC.
- This programme could be scaled up in other similar settings.
- There is a need to focus on designing, implementing, and evaluating multisectoral action plans and legislative measures in low income settings.

### Supplementary information

Supplementary Table 1

Supplementary Table 2

Supplementary Table 3

Supplementary Table 4

## Data Availability

The data used to support the findings of this study are available from the corresponding author upon request.
