# Supplementary material for: Effectiveness of a 3-year community-based intervention for blood pressure reduction among adults: a repeated cross-sectional study with a comparison area
Source: J Hum Hypertens. 2022 Apr 8;38(4):336–44. doi: 10.1038/s41371-022-00672-2 (PMC11001574; doi:10.1038/s41371-022-00672-2)
Supplement: Supplementary file 1 — Supplementary Table 1 [file 41371_2022_672_MOESM1_ESM.docx]

Supplemental Table 1: Change in of systolic blood pressure according to socio-demographic characteristics among adults before and after intervention in intervention and comparison areas in Sousse, Tunisia 2009-2014.

|  | | | | Intervention area | | | | | Comparison area | | | | |
| --- | --- | --- | --- | --- | --- | --- | --- | --- | --- | --- | --- | --- | --- |
| Systolic blood pressure (SBP) mmHg | | | | n | Baseline  m(sd)) | n | Follow up  m(sd) | p | n | Baseline  m(sd) | n | Follow up  m(sd) | p |
| **All participants m(sd) mmHg** | | | | 910 | 132.4(19.2) | 991 | 130.6(17.7) | 0.035 | 936 | 129.4(17.8) | 976 | 130.4(17.9) | 0.383 |
| **Gender** | | Male | | 385 | 135.1(17.9) | 439 | 134.2(17.0) | 0.442 | 269 | 131.9(16.8) | 335 | 134.2(16.8) | 0.087 |
|  |  | Female | | 525 | 130.4(19.9) | 552 | 127.8(17.8) | 0.022 | 667 | 128.8(18.1) | 641 | 128.4(18.2) | 0.689 |
| **Age groups** | | | <29 | 335 | 124.9(13.4) | 303 | 122.2(13.0) | 0.009 | 293 | 121.8(12.5) | 260 | 121.6(13.0) | 0.886 |
|  |  |  | [30-39] | 167 | 125.9(14.3) | 204 | 125.6(14.5) | 0.821 | 214 | 124.1(12.8) | 206 | 125.3(14.5) | 0.358 |
|  |  |  | [40-49] | 192 | 135.8(19.1) | 200 | 131.8(16.2) | 0.024 | 187 | 130.4(16.6) | 226 | 131.3(17.8) | 0.588 |
|  |  |  | ≤50 | 211 | 146.3(21.9) | 284 | 142.5(18.6) | 0.035 | 238 | 143.5(19.4) | 281 | 141.5(18.3) | 0.230 |
| **Educational level** | Illiterate or primary | | | 230 | 138.8(22.0) | 320 | 135.3(19.8) | 0.055 | 398 | 134.3(18.8) | 497 | 134.2(18.8) | 0.922 |
|  | College or secondary | | | 422 | 130.9(19.0) | 494 | 128.4(16.0) | 0.033 | 366 | 126.3(14.6) | 341 | 126.5(15.6) | 0.860 |
|  | University level | | | 258 | 129.1(15.4) | 166 | 127.7(16.1) | 0.348 | 171 | 126.0(15.5) | 137 | 126.1(17.2) | 0.917 |
| **Marital status** | | | Not married | 344 | 127.2(15.6) | 334 | 125.7(15.3) | 0.214 | 314 | 126.9(16.4) | 278 | 127.3(16.0) | 0.766 |
|  |  |  | Married | 563 | 135.6(20.5) | 657 | 133.1(18.3) | 0.026 | 621 | 131.1(18.3) | 689 | 131.6(18.5) | 0.632 |
| **Employment status** | | | Not working | 508 | 133.3(19.8) | 527 | 130.6(18.5) | 0.026 | 672 | 130.4(19.0) | 617 | 130.4(18.2) | 0.938 |
|  |  |  | working | 400 | 131.4(18.4) | 459 | 130.8(16.9) | 0.623 | 262 | 128.0(14.0) | 357 | 130.4(17.4) | 0.053 |
| **Employment status** | | | Not working | 77 | 128.9(16.6) | 155 | 126.7(15.9) | 0.331 | 92 | 129.3(16.1) | 143 | 131.3(19.8) | 0.422 |
|  |  |  | working | 400 | 131.4(18.4) | 459 | 130.8(16.9) | 0.623 | 262 | 127.9(14.0) | 357 | 130.4(17.4) | 0.061 |
|  |  |  | Student | 152 | 125.9(14.3) | 96 | 122.4(13.3) | 0.055 | 127 | 120.7(13.2) | 87 | 123.4(15.1) | 0.177 |
|  |  |  | Housewife | 238 | 136.1(20.7) | 223 | 132.4(18.9) | 0.047 | 415 | 131.9(19.4) | 342 | 130.7(17.7) | 0.359 |
|  |  |  | Retired | 41 | 151.9(22.6) | 53 | 149.1(17.8) | 0.506 | 38 | 148.1(21.3) | 45 | 139.6(18.8) | 0.058 |
| **Socio-economic level** | | | low | 45 | 133.6(18.1) | 18 | 126.6(16.8) | 0.164 | 19 | 129.1(21.7) | 35 | 127.0(11.4) | 0.641 |
|  |  |  | Low middle | 450 | 132.5(18.7) | 452 | 130.9(18.0) | 0.194 | 439 | 130.5(17.4) | 427 | 130.7(18.4) | 0.855 |
|  |  |  | High middle | 359 | 132.0(20.2) | 422 | 130.6(17.1) | 0.294 | 444 | 129.5(18.0) | 418 | 131.0(18.1) | 0.235 |
|  |  |  | High | 56 | 133.6(18.2) | 99 | 130.3(19.1) | 0.300 | 34 | 122.4(14.9) | 96 | 127.9(16.7) | 0.094 |
|  |  |  | No | 343 | 130.2(17.5) | 331 | 128.6(16.6) | 0.207 | 320 | 127.1(16.4) | 329 | 128.2(16.8) | 0.382 |
