# Supplementary material for: Effectiveness of a 3-year community-based intervention for blood pressure reduction among adults: a repeated cross-sectional study with a comparison area
Source: J Hum Hypertens. 2022 Apr 8;38(4):336–44. doi: 10.1038/s41371-022-00672-2 (PMC11001574; doi:10.1038/s41371-022-00672-2)
Supplement: Supplementary file 2 — Supplementary Table 2 [file 41371_2022_672_MOESM2_ESM.docx]

Supplemental Table 2: Change in systolic blood pressure according to high blood risk factors among adults before and after intervention in intervention and comparison areas in Sousse, Tunisia 2009-2014.

|  | | | Intervention area | | | | | Comparison area | | | | |
| --- | --- | --- | --- | --- | --- | --- | --- | --- | --- | --- | --- | --- |
| Systolic blood pressure (SBP) mmHg | | | n | Baseline  m(sd)) | n | Follow up  m(sd) | p | n | Baseline  m(sd) | n | Follow up  m(sd) | p |
| **Tobacco use** | | Yes | 229 | 134.1(18.7) | 229 | 133.1(17.9) | 0.546 | 133 | 129.8(16.1) | 178 | 133.3(16.0) | 0.058 |
|  |  | No | 666 | 131.9(19.4) | 761 | 129.9(17.4) | 0.043 | 802 | 129.7(18.0) | 797 | 128.9(18.3) | 0.917 |
| **Alcohol drinking** | | Yes | 76 | 134.7(18.0) | 90 | 132.0(17.7) | 0.348 | 26 | 125.6(07.4) | 27 | 131.9(18.5) | 0.111 |
|  |  | No | 834 | 132.2(19.3) | 895 | 130.5(17.7) | 0.063 | 910 | 129.8(18.0) | 932 | 130.3(17.9) | 0.569 |
| **Recommended physical activity** | | Yes | 490 | 130.3(17.7) | 546 | 129.4(17.0) | 0.437 | 505 | 127.9(16.4) | 588 | 129.5(17.7) | 0.138 |
|  |  | No | 420 | 134.9(20.6) | 445 | 132.1(18.5) | 0.035 | 431 | 131.8(19.1) | 388 | 131.8(18.2) | 0.959 |
| **Sedentary** | | Yes | 785 | 132.7(18.9) | 879 | 130.5(17.7) | 0.015 | 889 | 129.5(17.8) | 878 | 130.6(18.1) | 0.194 |
|  |  | No | 122 | 131.1(21.3) | 105 | 131.4(17.6) | 0.919 | 44 | 133.6(17.6) | 96 | 128.8(15.8) | 0.112 |
| **five or more servings per day** | | Yes | 296 | 132.7(18.4) | 495 | 131.7(17.4) | 0.460 | 459 | 128.7(17.8) | 678 | 131.6(18.0) | 0.007 |
|  |  | No | 610 | 132.3(19.7) | 489 | 129.5(18.0) | 0.015 | 474 | 130.8(17.6) | 295 | 127.8(17.4) | 0.022 |
| **Weight status** | Normal weight | | 353 | 126.2(15.5) | 342 | 124.8(15.6) | 0.244 | 362 | 122.3(14.0) | 334 | 124.4(15.7) | 0.061 |
|  | overweight | | 303 | 135.0(19.9) | 348 | 131.5(17.6) | 0.021 | 292 | 131.3(17.3) | 308 | 130.8(17.8) | 0.694 |
|  | obesity | | 239 | 138.4(20.9) | 294 | 136.1(18.0) | 0.179 | 270 | 137.9(18.8) | 324 | 136.2(18.2) | 0.263 |
| **Central obesity** | | Yes | 397 | 137.6(21.7) | 451 | 133.7(18.8) | 0.006 | 466 | 134.0(18.8) | 469 | 134.3(18.5) | 0.778 |
|  |  | No | 507 | 128.6(15.8) | 532 | 128.2(16.4) | 0.674 | 464 | 125.3(15.5) | 503 | 126.7(16.6) | 0.154 |
| **Add salt to meal** | | Yes | 241 | 127.3(16.4) | 217 | 127.3(16.4) | 0.951 | 147 | 124.9(17.1) | 114 | 126.8(17.7) | 0.381 |
|  | | No | 666 | 134.3(19.9) | 773 | 131.5(17.9) | 0.006 | 788 | 130.6(17.8) | 861 | 130.9(17.9) | 0.755 |
| **Diagnosed hypertension** | | Yes | 70 | 157.7(23.8) | 87 | 149.3(19.6) | 0.016 | 97 | 152.5(19.6) | 109 | 148.7(20.6) | 0.172 |
|  |  | No | 840 | 130.3(17.2) | 904 | 128.8(16.4) | 0.068 | 839 | 127.1(15.5) | 867 | 128.1(16.2) | 0.169 |
| **Treated hypertension** | | Yes | 54 | 159.1(24.0) | 74 | 148.8(19.1) | 0.008 | 80 | 154.4(19.6) | 88 | 149.6(21.1) | 0.129 |
|  |  | No | 59 | 145.4(20.7) | 77 | 137.1(16.9) | 0.012 | 75 | 137.2(18.2) | 152 | 132.6(17.2) | 0.065 |
| **Family history of hypertension** | | Yes | 567 | 133.7(20.1) | 660 | 131.7(18.2) | 0.058 | 616 | 131.1(18.3) | 646 | 131.5(18.4) | 0.645 |
|  |  | No | 343 | 130.2(17.5) | 331 | 128.6(16.6) | 0.207 | 320 | 127.1(16.4) | 329 | 128.2(16.8) | 0.382 |
