# Supplementary material for: Effectiveness of a 3-year community-based intervention for blood pressure reduction among adults: a repeated cross-sectional study with a comparison area
Source: J Hum Hypertens. 2022 Apr 8;38(4):336–44. doi: 10.1038/s41371-022-00672-2 (PMC11001574; doi:10.1038/s41371-022-00672-2)
Supplement: Supplementary file 3 — Supplementary Table 3 [file 41371_2022_672_MOESM3_ESM.docx]

Supplemental Table 3: Change in diastolic blood pressure according to socio-demographic characteristics among adults before and after intervention in intervention and comparison areas in Sousse, Tunisia 2009-2014

|  | | Intervention area | | | | | Comparison area | | | | |
| --- | --- | --- | --- | --- | --- | --- | --- | --- | --- | --- | --- |
| Diastolic blood pressure (DBP) mmHg | | n | Baseline  m(sd) | n | Follow up  m(sd) | p | n | Baseline  m(sd) | n | Follow up  m(sd) | p |
| **All participants** |  | 909 | 78.7(11.8) | 991 | 76.9(11.1) | <0.001 | 935 | 78.1(10.8) | 976 | 76.8(11.0) | 0.007 |
| **Gender** | Male | 385 | 79.2(12.2) | 436 | 78.0(11.1) | 0.160 | 268 | 78.1(10.5) | 335 | 77.8(11.5) | 0.734 |
|  | Female | 524 | 78.4(11.5) | 552 | 76.0(11.0) | <0.001 | 667 | 78.1(10.9) | 641 | 76.2(10.6) | 0.002 |
| **Age groups** | <29 | 335 | 75.2(10.8) | 303 | 72.0(09.9) | <0.001 | 292 | 73.6(09.6) | 260 | 72.5(10.4) | 0.230 |
|  | [30-39] | 167 | 77.0(09.9) | 204 | 76.9(11.0) | 0.942 | 214 | 78.3(09.5) | 206 | 75.2(10.2) | 0.001 |
|  | [40-49] | 191 | 81.4(12.2) | 200 | 78.9(10.6) | 0.033 | 187 | 79.5(10.5) | 226 | 79.4(10.2) | 0.961 |
|  | ≤50 | 211 | 83.4(12.0) | 284 | 80.6(10.8) | 0.009 | 238 | 82.4(11.4) | 281 | 79.5(11.1) | 0.004 |
| **Educational level** | Illiterate or primary | 229 | 80.9(11.6) | 329 | 78.9(11.7) | 0.039 | 398 | 80.5(11.1) | 497 | 77.9(11.0) | <0.001 |
|  | College or secondary | 422 | 78.2(11.9) | 494 | 75.8(10.8) | 0.001 | 365 | 76.1(10.6) | 341 | 75.9(10.7) | 0.762 |
|  | University level | 258 | 77.6(11.4) | 166 | 76.3(10.2) | 0.205 | 171 | 76.7(09.3) | 137 | 74.6(11.1) | 0.077 |
| **Marital status** | Not married | 344 | 76.6(11.0) | 334 | 73.9(10.5) | 0.001 | 313 | 76.2(10.2) | 278 | 74.2(10.8) | 0.027 |
|  | Married | 621 | 79.1(10.9) | 689 | 77.7(10.8) | 0.021 | 562 | 80.1(12.0) | 657 | 78.4(11.1) | 0.013 |
| **Employment status** | Not working | 508 | 78.9(12.1) | 527 | 76.3(11.0) | <0.001 | 671 | 78.5(11.1) | 617 | 76.6(10.6) | 0.002 |
|  | working | 399 | 78.6(11.3) | 459 | 77.5(11.2) | 0.163 | 262 | 77.3(09.9) | 357 | 77.1(11.6) | 0.772 |
| **Employment status** | Not working | 77 | 77.2(12.3) | 155 | 74.7(10.2) | 0.131 | 92 | 79.9(10.4) | 143 | 77.8(11.2) | 0.155 |
|  | working | 399 | 78.6(11.3) | 459 | 77.5(11.2) | 0.163 | 262 | 77.3(09.9) | 357 | 77.1(11.6) | 0.772 |
|  | Student | 152 | 75.0(10.7) | 96 | 72.9(10.5) | 0.132 | 126 | 73.0(08.9) | 87 | 73.6(10.8) | 0.677 |
|  | Housewife | 238 | 81.1(11.8) | 223 | 77.9(11.4) | 0.004 | 415 | 79.1(11.3) | 342 | 76.5(10.2) | 0.001 |
|  | Retired | 41 | 83.3(14.0) | 53 | 80.5(10.3) | 0.262 | 38 | 86.0(09.8) | 45 | 79.5(10.9) | 0.006 |
| **Socio-economic level** | low | 45 | 82.4(13.9) | 18 | 72.1(10.2) | 0.006 | 19 | 73.3(08.1) | 35 | 76.9(09.2) | 0.158 |
|  | Low middle | 449 | 78.9(12.1) | 452 | 77.6(11.2) | 0.097 | 439 | 79.0(10.8) | 427 | 76.8(10.3) | 0.001 |
|  | High middle | 359 | 78.0(11.2) | 422 | 76.3(10.9) | 0.034 | 444 | 77.6(10.8) | 418 | 76.9(11.8) | 0.384 |
|  | High | 56 | 79.4(09.7) | 99 | 77.1(11.2) | 0.202 | 33 | 75.6(10.3) | 96 | 76.0(11.0) | 0.848 |
