# Supplementary material for: Effectiveness of a 3-year community-based intervention for blood pressure reduction among adults: a repeated cross-sectional study with a comparison area
Source: J Hum Hypertens. 2022 Apr 8;38(4):336–44. doi: 10.1038/s41371-022-00672-2 (PMC11001574; doi:10.1038/s41371-022-00672-2)
Supplement: Supplementary file 4 — Supplementary Table 4 [file 41371_2022_672_MOESM4_ESM.docx]

Supplemental Table 4: Change in diastolic blood pressure according to high blood risk factors among adults before and after intervention in intervention and comparison areas in Sousse, Tunisia 2009-2014.

|  | | | | Intervention area | | | | | | Comparison area | | | | |
| --- | --- | --- | --- | --- | --- | --- | --- | --- | --- | --- | --- | --- | --- | --- |
| Diastolic blood pressure (DBP) mmHg | | | | n | Baseline  m(sd) | n | Follow up  m(sd) | | p | n | Baseline  m(sd) | n | Follow up  m(sd) | p |
| **Tobacco use** | Yes | | | 229 | 78.5(12.2) | 229 | 77.5(11.0) | | 0.341 | 133 | 77.0(10.2) | 178 | 77.0(11.2) | 0.987 |
|  | No | | | 665 | 78.9(11.5) | 761 | 76.7(11.1) | | <0.001 | 801 | 78.3(10.8) | 797 | 76.2(10.9) | 0.003 |
| **Alcohol drinking** | Yes | | | 76 | 78.0(11.6) | 90 | 77.8(11.7) | | 0.921 | 26 | 74.7(11.2) | 27 | 75.6(12.2) | 0.768 |
|  | No | | | 833 | 78.8(11.8) | 895 | 76.9(11.0) | | <0.001 | 909 | 78.2(10.7) | 932 | 76.8(11.0) | 0.006 |
| **Recommended physical activity** | | | Yes | 489 | 78.1(11.2) | 546 | | 76.3(10.9) | 0.009 | 505 | 77.8(10.5) | 588 | 76.6(11.0) | 0.089 |
|  |  |  | No | 420 | 79.5(12.4) | 445 | | 77.6(11.3) | 0.021 | 430 | 78.5(11.0) | 388 | 76.9(10.9) | 0.038 |
| **sedentary** | | | Yes | 784 | 78.9(11.6) | 879 | | 76.7(11.1) | <0.001 | 888 | 78.1(10.8) | 878 | 76.7(11.0) | 0.009 |
|  |  |  | No | 122 | 77.6(12.7) | 105 | | 78.2(11.3) | 0.701 | 44 | 79.5(10.6) | 96 | 77.1(10.4) | 0.207 |
| **five or more servings per day** | | | Yes | 296 | 78.8(11.2) | 495 | | 77.8(10.7) | 0.202 | 459 | 77.7(10.9) | 678 | 77.2(11.3) | 0.444 |
|  |  |  | No | 609 | 78.7(12.0) | 489 | | 75.9(11.3) | <0.001 | 474 | 78.5(10.6) | 295 | 75.8(10.1) | 0.001 |
| **Weight status** | | Normal weight | | 353 | 75.4(11.1) | 342 | | 73.6(10.0) | 0.022 | 362 | 74.3(09.6) | 334 | 73.7(10.7) | 0.466 |
|  | | overweight | | 303 | 80.0(11.7) | 348 | | 77.7(11.1) | 0.011 | 291 | 78.5(10.0) | 308 | 76.9(19.5) | 0.058 |
|  | | obesity | | 238 | 82.1(11.8) | 294 | | 79.8(11.4) | 0.025 | 270 | 83.0(11.1) | 324 | 79.8(11.5) | 0.001 |
| **Central obesity** | | | Yes | 396 | 81.2(12.1) | 451 | | 78.9(11.3) | 0.005 | 466 | 80.5(11.0) | 469 | 78.6(10.9) | 0.008 |
|  | | | No | 507 | 76.9(11.1) | 532 | | 75.2(10.7) | 0.018 | 463 | 75.8(10.0) | 503 | 75.0(10.7) | 0.297 |
| **Add salt to meals** | | | Yes | 241 | 75.9(10.4) | 217 | | 75.2(11.2) | 0.514 | 147 | 75.7(10.6) | 114 | 75.6(11.7) | 0.951 |
|  | | | No | 665 | 79.8(12.1) | 773 | | 77.4(11.0) | <0.001 | 787 | 78.6(10.8) | 861 | 76.9(10.8) | 0.002 |
| **Diagnosed hypertension** | | | Yes | 70 | 86.7(13.2) | 87 | | 83.3(11.3) | 0.085 | 97 | 85.0(12.7) | 109 | 82.1(12.2) | 0.099 |
|  |  |  | No | 839 | 78.1(11.4) | 904 | | 76.3(10.9) | 0.001 | 838 | 77.3(10.2) | 867 | 76.1(10.6) | 0.015 |
| **Treated hypertension** | | | Yes | 80 | 84.5(11.9) | 88 | | 81.3(12.7) | 0.090 | 54 | 86.5(13.6) | 74 | 82.6(10.9) | 0.078 |
|  |  |  | No | 59 | 84.0(12.4) | 77 | | 79.5(11.8) | 0.032 | 75 | 83.6(12.3) | 152 | 77.6(09.5) | <0.001 |
| **Family history of hypertension** | | | Yes | 567 | 79.3(11.9) | 660 | | 77.6(11.3) | 0.011 | 616 | 78.7(10.8) | 646 | 77.6(11.2) | 0;059 |
|  |  |  | No | 342 | 77.7(11.5) | 331 | | 75.4(10.5) | 0.006 | 319 | 74.9(10.6) | 329 | 75.2(10.2) | 0.036 |
